# The Role of Cytokines in Neutrophil Development, Tissue Homing, Function and Plasticity in Health and Disease

**DOI:** 10.3390/cells12151981

**Published:** 2023-07-31

**Authors:** Maria Tsioumpekou, Daniëlle Krijgsman, Jeanette H. W. Leusen, Patricia A. Olofsen

**Affiliations:** 1 Center for Translational Immunology, University Medical Center Utrecht, 3584 CX Utrecht, The Netherlands; m.tsioumpekou@umcutrecht.nl (M.T.); d.krijgsman-4@umcutrecht.nl (D.K.); jleusen@umcutrecht.nl (J.H.W.L.); 2 Center for Molecular Medicine, University Medical Center Utrecht, 3584 CX Utrecht, The Netherlands

**Keywords:** neutrophils, cytokines, tissue-resident neutrophils, autoimmune diseases, cancer, tumor microenvironment, TME, NETs, cytokine therapeutics, immunocytokines, immunotherapy

## Abstract

Neutrophils are crucial innate immune cells and comprise 50–70% of the white blood cell population under homeostatic conditions. Upon infection and in cancer, blood neutrophil numbers significantly increase because of the secretion of various chemo- and cytokines by, e.g., leukocytes, pericytes, fibroblasts and endothelial cells present in the inflamed tissue or in the tumor microenvironment (TME). The function of neutrophils in cancer has recently gained considerable attention, as they can exert both pro- and anti-tumorigenic functions, dependent on the cytokine milieu present in the TME. Here, we review the effect of cytokines on neutrophil development, tissue homing, function and plasticity in cancer and autoimmune diseases as well as under physiological conditions in the bone marrow, bloodstream and various organs like the spleen, kidney, liver, lung and lymph nodes. In addition, we address several promising therapeutic options, such as cytokine therapy, immunocytokines and immunotherapy, which aim to exploit the anti-tumorigenic potential of neutrophils in cancer treatment or block excessive neutrophil-mediated inflammation in autoimmune diseases.

## 1. Introduction

**Figure 1:**
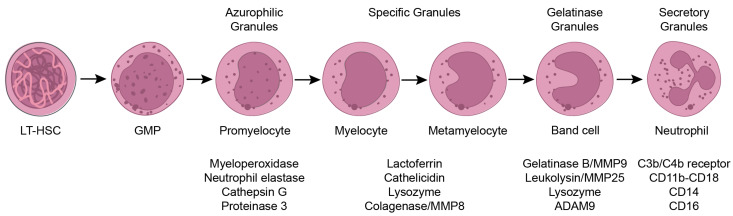
Neutrophil development in the bone marrow. Long-term hematopoietic stem cells give rise to mature neutrophils via several stem and progenitor cell stages, promyelocytes, myelocytes, metamyelocytes and band cells. Granule content differs between various stages of differentiation and comprises proteins like neutrophil elastase, collagenase and gelatinase. LT-HSC: long-term hematopoietic stem cell; GMP: granulocyte–monocyte progenitor; MMP: matrix metalloproteinase.

**Figure 2:**
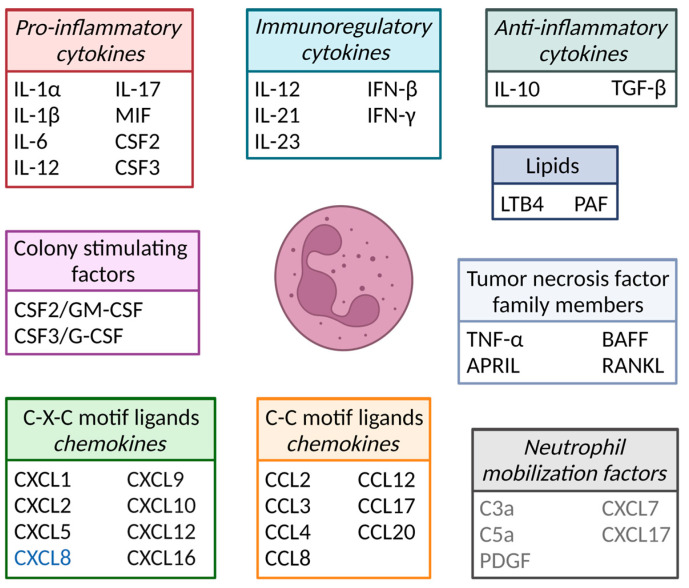
Cytokines and chemokines produced by neutrophils and/or other cells that affect neutrophil function, which will be discussed in this review. IL = interleukin; MIF = macrophage migration inhibitory factor; CSF = colony-stimulating factor; IFN = interferon; TGF = transforming growth factor; LTB4 = leukotriene B4; PAF = platelet-activating factor; CXCL = C-X-C motif ligand; CCL = C-C motif ligand; TNF = tumor necrosis factor; APRIL = a proliferation-inducing ligand; BAFF = B cell-activating factor; RANKL = receptor activator of NF-κB ligand; C3a/C5a = complement factor 3a/5a; PDGF = platelet-derived growth factor. Of note, the *CXCL8* gene (indicated in blue) is lacking in mice, and the neutrophil mobilization factors (shown in gray) are not produced by neutrophils but do affect neutrophil mobilization.

Neutrophils are the body’s first line of defense against pathogens, e.g., bacteria and fungi, and comprise 50–70% of the white blood cell population in human circulation. They are essential immune cells, and patients that lack (mature) neutrophils often succumb to severe opportunistic bacterial infections [1]. Neutrophils contain at least four types of granules: azurophilic/primary-, specific/secondary-, gelatinase/tertiary-, and secretory-granules and are, therefore, together with eosinophils and basophils, also known as granulocytes [2]. The different classes of granules are formed sequentially during neutrophil differentiation and contain different proteins important to pathogen killing (Figure 1) [2]. In addition to granule proteins, neutrophils also produce various cytokines and chemokines important for, e.g., pathogen killing and the attraction of leukocytes, respectively (Figure 2). Cytokines comprise a large group of secreted pro- and anti-inflammatory factors, grouped based on their structural homology, the similarity of their receptors and/or function (Figure 2). Chemokines are a subgroup of cytokines whose generic function is to induce cell migration.

Neutrophils have a short half-life in blood, ranging from 13 to 19 hours under homeostatic conditions [3]. Given their rapid turnover, approximately 1 billion neutrophils per kilogram of body weight are produced daily, which can be extended to 10 billion under disease conditions, e.g., inflammation and cancer [4,5,6]. For a long time, it was believed that neutrophils were specialized cells that existed to prevent infections and could not be more versatile because of their short half-life. However, since several reports showed the prominent pro- and anti-tumorigenic roles of neutrophils in cancer, they have gained increased attention [7,8,9,10]. In this review, we will discuss neutrophil production, function and plasticity, with an emphasis on the role cytokines play in these processes, and describe potential strategies to exploit the anti-tumorigenic potential of neutrophils, as well as strategies to block excessive neutrophil-mediated inflammation in autoimmune diseases.

## 2. Neutrophils in the Bone Marrow

Neutrophils are derived from long-term hematopoietic stem cells (LT-HSCs) in the bone marrow and, via several stem and (multipotent) progenitor cell stages, differentiate into granulocyte–monocyte progenitors (GMPs) [11,12]. These GMPs give rise to mature neutrophils via promyelocyte, myelocyte, metamyelocyte and banded neutrophil stages (Figure 1). This differentiation process is controlled by colony-stimulating factor 3 (CSF3), better known as granulocyte colony-stimulating factor (G-CSF). CSF3 is not only involved in neutrophil differentiation but also plays a key role in the release of neutrophils from the bone marrow into the circulation. CSF3 signaling results in the downregulation of CXCL12 and its receptor CXCR4, which are essential for neutrophil retention in the bone marrow [13,14]. Mice lacking *Csf3* or its receptor (*Csf3r*) are severely neutropenic, indicating that this signaling pathway is essential for normal neutrophil production and release [15,16]. In addition to data obtained from genetically altered mice, mutations in the *CSF3R* gene are found in humans and result in severe congenital neutropenia, characterized by low absolute neutrophil counts (<0.5 × 10^9^/L) in the circulation [17]. Activating mutations of CXCR4 are also found in humans, resulting in neutrophil accumulation in the bone marrow and neutropenia in the circulation, in a disease known as WHIM syndrome [18].

In addition to CSF3, colony-stimulating factor 2 (CSF2), also known as granulocyte–macrophage colony-stimulating factor (GM-CSF), and interleukin-6 (IL-6) are described as being involved in the production of neutrophils, especially during inflammatory responses in a process called emergency granulopoiesis (described in more detail in Section 7. Neutrophils in Severe Infection and Inflammation) [19,20,21].

## 3. Circulating Neutrophils

Once neutrophils are released from the bone marrow, they enter the circulation, where they can stay up to 19 hours [3]. Because of the rapid turnover of circulating neutrophils, they have long been viewed as a homogeneous population. However, the density gradient separation of peripheral blood using Ficoll has identified neutrophils in both the high-density granulocytic fraction and the low-density mononuclear cell fraction, suggesting some degree of functional or structural stratification [22]. In healthy humans and mice, this low-density neutrophil (LDN) population is negligible, but it increases with tumor growth. Follow-up studies have determined that LDN consists of both a mature and an immature population, possibly caused by different degranulation and/or maturation states [23]. In addition to an altered phenotype, increased levels of blood neutrophils have been associated with poor disease outcomes in advanced cancer patients, and the neutrophil-to-lymphocyte ratio is used as a prognostic factor in many tumor types [24,25].

In recent years, striking new insights have been gained about circulating neutrophil subsets using new techniques, e.g., single-cell RNA sequencing and single-cell mass cytometry by time-of-flight (CyTOF). Zilionis et al. described the identification of six different circulating neutrophil subsets (termed N1 to N6) based on single-cell RNA sequencing blood from six treatment-naïve patients with non-small-cell lung cancer [26]. Whether all six transcriptionally defined subsets can be found in healthy individuals or whether some subsets are cancer-associated remains to be determined, but what is clear is that neutrophils are more diverse than previously thought.

In addition to the subdivision of circulating neutrophils based on their transcriptomes, Zhu and colleagues used a CyTOF panel containing 40 of the most commonly used surface markers of neutrophil maturation, activation and function to investigate neutrophil subsets in blood from 21 treatment-naïve patients with melanoma [27]. In addition to a neutrophil progenitor (hNeP) population, they identified six neutrophil clusters (Cneut1 to Cneut6), each with a distinct surface marker expression. Significant variations in subset frequencies were observed when comparing the data from the blood of melanoma patients with blood samples from two healthy controls. The largest neutrophil subset, the terminally differentiated mature neutrophil, Cneut2, decreased from >95% of the total neutrophil population in healthy donors to <90% in patients with melanoma, while the hNeP subset significantly increased. This is in line with previous data, where part of the tumor-associated circulating LDN is described as displaying immature characteristics [28].

## 4. Neutrophil Extravasation

**Figure 3:**
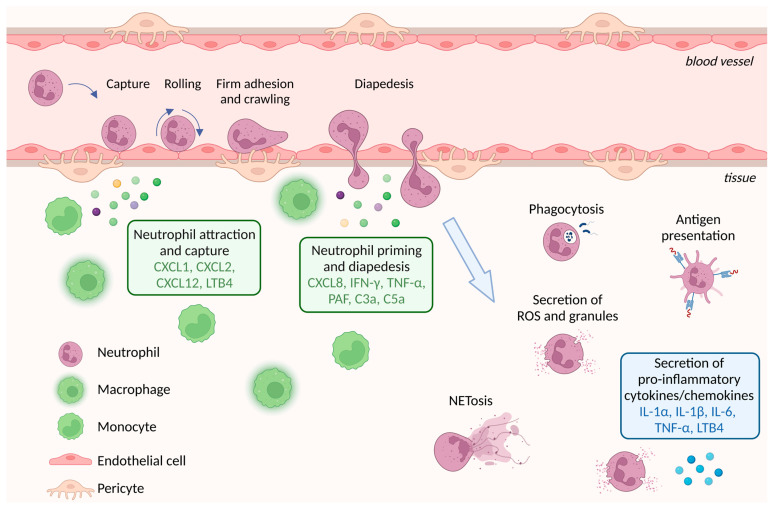
Neutrophil extravasation. Chemokines produced by, e.g., monocytes and macrophages, attract neutrophils to the vessel wall and mediate capture, rolling, adhesion and diapedesis into the inflamed tissue. In the tissue, neutrophils can perform phagocytosis and NETosis; secrete ROS, granules and pro-inflammatory cytokines; and present antigens.

As first responders upon microbial infection or injury, neutrophils rapidly migrate into affected tissues to regulate pathogen dissemination and contribute to the resolution of inflammation. Neutrophil extravasation and emigration to infected sites are complex multi-step cascades, highly dependent on the interplay between neutrophils and various cell types, such as endothelial cells, perivascular cells and stromal cells (Figure 3) [29,30,31]. Specifically, chemoattractants, such as chemokine C-X-C motif ligand 1 (CXCL1), CXCL2, CXCL12 and leukotriene B4 (LTB4), are secreted by activated perivascular leukocytes and induce neutrophil capture by endothelial cells and rolling along the vascular wall (Figure 3). Neutrophil priming then occurs, mediated by CXCL8; interferon gamma (IFN-γ); tumor necrosis factor-alpha (TNF-α); platelet-activating factor (PAF); complement factors C3a and C5a; and/or bacterial peptides, leading to firm adhesion to the endothelial cells and eventual transendothelial migration (diapedesis) through the pericyte layer and basement membrane (Figure 3) [31,32,33]. Extravasated neutrophils migrate toward the infected site following a gradient of chemokines secreted by tissue-resident cells, where they produce reactive oxygen species (ROS), phagocytose bacteria or neutrophil extracellular traps (NETs) in a process called NETosis (Figure 3) [34]. NETs are composed of chromatin (DNA and histones) fibers associated with various antimicrobial proteins. NETs can trap and kill microbes; however, their excessive or dysregulated production can also contribute to tissue damage and inflammation, including autoimmune disorders and cancer (discussed in more detail later on in this review). Although neutrophils were long considered to be devoid of significant transcriptional activity, it is now accepted that they are capable of the de novo production and release of various cytokines and chemokines, e.g., pro-inflammatory cytokines (IL-1α, IL-1β, IL-6 and TNF-α), anti-inflammatory cytokines and leukotrienes, which affect leukocyte attraction and activation as well as the enhancement or resolution of inflammation, among others [35,36]. Neutrophil migration into tissues has been investigated thoroughly over the last few decades, and it has become evident that the mechanisms underlying it are organ-specific and dependent on inflammatory stimuli [37]. Structural specializations; variations in the tissue microenvironment and/or neutrophil priming; and the activation state prior to reaching the tissue, as well as the differential expression of the molecules involved, can contribute to this specificity, and understanding these differences is crucial for future therapeutic interventions.

## 5. Tissue-Resident Neutrophils

**Figure 4:**
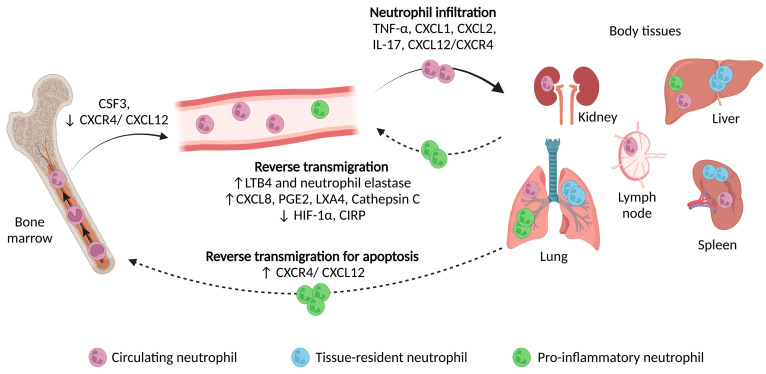
Tissue infiltration and reverse transmigration of neutrophils. Following their generation in the bone marrow, neutrophils are released into the bloodstream through a CSF3-induced decrease in CXCR4/CXCL12 signaling. Under homeostatic conditions, neutrophils routinely patrol different organs to limit the growth of the commensal biofilm. Upon injury or infection and subsequent cytokine secretion, neutrophils are actively recruited into the tissue to perform their effector functions. Pro-inflammatory neutrophils have also been shown to exit the damaged tissue and re-enter the circulation. Via reverse transmigration, neutrophils can either migrate to other organs and lead to the dissemination of inflammation or age and return to the bone marrow to finally undergo apoptosis.

Research on neutrophil recruitment into the lungs, spleen, lymph nodes, kidneys and other organs has highlighted the existence of different neutrophil subpopulations with distinct functions. Interestingly, the presence of neutrophils in these organs occurs not only upon infection or inflammation but also under homeostatic conditions. Marginated pools of neutrophils that adhere to the endothelium and serve as a reservoir upon stimulation have been identified in the liver, spleen and lungs (although more controversially in the latter). The biodistribution of neutrophils is dependent on their maturation and activation state and is, among other things, affected by microvascular blood flow and factors such as exercise, drugs and infection [38,39,40,41]. Apart from circulatory and marginated neutrophils, studies have also highlighted the existence of resident neutrophils patrolling healthy tissue matrices, especially at mucosal sites, including the gastrointestinal, respiratory, reproductive and ocular mucosa, where there is a constitutive microbial biofilm (Figure 4) [42,43].

### 5.1. Neutrophils in the Spleen

Puga et al. were the first to identify two neutrophil subpopulations residing in the marginal zone (MZ) of the spleen in mice, which showed a different marker profile compared with circulating neutrophils. They demonstrated that IL-10 secreted by sinusoidal endothelial cells upon microbial TLR signaling would reprogram neutrophils residing in the spleen into B helper neutrophils. This novel neutrophil subset would then activate MZ B cells by secreting B cell-activating factor (BAFF), a proliferation-inducing ligand (APRIL) and IL-21, thus inducing Ig class switching, somatic hypermutation and antibody production [44]. The existence of MZ B helper neutrophils was later verified in mice via intravital imaging by Deniset et al., who additionally discovered two neutrophil subpopulations (Ly6G^int^ immature and Ly6G^high^ mature) in the red pulp (RP) of the spleen both under steady state and upon *S. pneumoniae* infection. Upon infection, the mature neutrophils played an immune surveillance role and facilitated bacterial clearance along with RP macrophages, whereas the immature neutrophils served as a reservoir in case of emergency [45].

### 5.2. Neutrophils in the Kidneys

Single-cell sequencing studies have provided new insights into the presence and heterogeneity of neutrophils in kidneys in both healthy and pathological conditions [46,47,48]. Particularly, the transcriptional profiling of kidney biopsies from healthy controls and clear-cell renal carcinoma (ccRCC) patients revealed the presence of two neutrophil subpopulations in a healthy kidney, one related to renal autoimmunity and another providing protection against infections, whereas six subpopulations were identified in ccRCC patient kidneys [49]. Although the mechanisms underlying neutrophil recruitment in the kidney remain quite understudied, it is now known that it can occur in all three distinct capillary networks found in this organ and that various proteins, e.g., P-selectin, E-selectin, intercellular adhesion molecule 1 (ICAM-1), β2-integrins and P-selectin glycoprotein ligand-1 (PSGL-1), can be of importance depending on the exact location [40]. Furthermore, upon infection with Shiga-toxin-producing enterohemorrhagic *E. coli*, neutrophil recruitment was dependent on TNF-α, CXCL1 and CXCL2 produced by tissue-resident macrophages and was directly associated with kidney injury and poor disease outcomes [50]. This is not the first study highlighting the contradictory role of neutrophils, as their presence in kidneys has been extensively correlated with poor prognosis for patients suffering from acute kidney injury, renal cancer, diabetic kidney disease and renal failure [51,52,53].

### 5.3. Neutrophils in the Liver

The dual role of neutrophils has also been investigated in liver tissues. Although the routine patrolling of neutrophils in liver sinusoids fortifies the liver upon infection, increased hepatic infiltration is also a key feature of most liver pathologies. Neutrophils are able to contribute to liver regeneration following hepatectomy by promoting the Kupffer cell/tissue-resident macrophage-dependent secretion of IL-6 and TNF-α. However, their excessive secretion of ROS and cytokines such as IL-1β, TNF-α, transforming growth factor beta (TGF-β) and IL-17, as well as their degranulation and NET formation, can aggravate the liver upon injury, ischemia-reperfusion, cirrhosis, fibrosis and cancer [54,55].

### 5.4. Neutrophils in the Lungs

Owing to the COVID-19 pandemic, the role of neutrophils in the lung has gained increasing attention and heightened research conducted in this area. Excessive neutrophil infiltration in the lung is considered a hallmark of acute respiratory distress disease (ARDS), observed in 29–42% of COVID patients, often resulting in fatality [56,57]. Under physiological conditions, the lung possesses a marginated pool of neutrophils. These neutrophils serve as a reservoir and are the first responders against the various pathogens and allergens that constantly enter the airways and make the lungs prone to inflammation [41]. The small size of the capillaries; the unique anatomical architecture of the lung with its bronchial and pulmonary vasculature; and the expression of CXCL12 by lung endothelial cells, which binds to CXCR4 on the neutrophils, contribute to the retainment of neutrophils in the lungs [40,58]. Upon infection, chemoattractants such as CXCL1, CXCL2 and IL-17 are produced in the lung, leading to further neutrophil recruitment and transmigration into the tissue, a process that is highly dependent on C-C chemokine receptor type 2 (CCR2)-positive blood monocytes [59,60]. Specifically, an RNA-sequencing analysis of CCR2^+^ monocytes recruited in cystic fibrosis airways showed a skewed pro-inflammatory profile with an increased expression of cytokines, e.g., *Cxcl1*, *Cxcl2* and *Csf3*, known to drive neutrophil chemotaxis and differentiation [61]. Additionally, intravital imaging revealed that, upon the depletion of monocytes using clodronate liposomes, neutrophil extravasation into the lung was severely reduced [59]. Following recruitment, neutrophils activate and elicit their effector functions, and their activation status can significantly differ based on the levels of chemokines and cytokines present. This has been especially highlighted in severe COVID cases, where a cytokine storm, characterized by elevated levels of IL-1β, IL-2, IL-6, IL-7, CXCL8, IL-10, IL-17, IFN-γ, IFN-γ-inducible protein 10 (IP-10/CXCL10), monocyte chemoattractant protein 1 (MCP1/CCL2), CSF3, macrophage inflammatory protein 1α (MIP-1a, also known as CCL3) and TNF-α was linked with increased neutrophil infiltration, NETosis, ROS production, thrombosis and mortality [56,57,62,63,64]. Interestingly, these observations are not restricted to SARS-CoV-19 infection, as excessive neutrophil recruitment in the lungs has been related to age-associated increases in influenza mortality [65]. Furthermore, single-cell analyses of non-small-cell lung cancer (NSCLC) patient samples have illustrated that neutrophils are the dominant immune cell type in the tumor microenvironment (TME) and found a correlation between neutrophil abundance and tumor heterogeneity, further highlighting the need for therapeutic manipulation of neutrophils in cancer [66,67,68].

### 5.5. Neutrophils in Lymph Nodes and Neutrophil–Dendritic Cell Hybrids

In recent years, a lot of attention has been drawn to the recruitment and role of neutrophils in the lymph nodes. Neutrophils routinely patrol the lymph nodes during steady-state conditions, and upon infection, they recruit additional neutrophils by releasing LTB4 [69]. As revealed by intravital imaging, the migration of neutrophils into the draining lymph nodes occurs both via high endothelial venules (HEVs), in a manner similar to lymphocytes, and via lymphatic vessels. Different molecules are involved based on the route of migration, i.e., HEV entry is L-selectin-; lymphocyte function-associated antigen 1 (LFA-1)-; very late antigen-4 (VLA-4)-; and C5a-dependent, whereas entry via efferent lymphatics highly depends on ICAM-1, CD11b, CXCR4, matrix metalloproteinases (MMPs) and occasionally CCR7 [70,71,72]. Based on the stimulus, additional cytokines may be involved in neutrophil recruitment into the lymph nodes, as IL-1β and IL-17 have also been suggested to mediate neutrophil migration upon injecting Vaccinia Virus Ankara and local tumor lysis, respectively [73,74]. Following challenges with *P. aeruginosa*, *S. aureus* and *Salmonella enterica* or the injection of Bacillus Calmette–Guérin (BCG), neutrophils have been shown to localize in different zones of the lymph nodes and be in close proximity to T and B cells, as well as innate-like lymphocytes such as γδ T cells, natural killer (NK) cells and natural killer T (NKT) cells [75,76,77,78]. This observation raised questions regarding the role of neutrophils in the lymph nodes and possible interactions with other immune cell types. In addition to their primary role in pathogen killing, lymph node neutrophils have been discovered to have additional functions. They have the ability to positively regulate leukocyte recruitment by secreting CCL3 and attracting dendritic cells (DCs) following *L. major* infection. Conversely, they can also negatively impact the immune response by facilitating the removal of subcapsular sinus macrophages during parasite infection [70]. Their ability to transfer antigens and stimulate adaptive immune responses can also be exploited by pathogens, as neutrophils can serve as “Trojan horses”, facilitating bacterial dissemination [70,78,79,80]. Although more research is needed on the role of cytokines in this process, CXCL8 and CXCL2 have been shown to mediate the uptake and intracellular survival of pathogenic bacterial strains (*S. aureus* and *Leishmania major*) in human and mouse neutrophils, respectively [81,82]. Infection with these bacterial or protozoan strains delays neutrophil apoptosis, resulting in an increased lifespan of up to 2–3 days, in which they release the monocyte attractant MIP-1β/CCL4 [82]. The uptake of apoptotic neutrophils by recruited monocytes/macrophages silences their antimicrobial functions, resulting in parasite survival and multiplication, followed by disease development [82,83,84]. The modulation of adaptive immunity by neutrophils has been extensively investigated and debated. Several research groups have reported the existence of a neutrophil subset with antigen presentation capabilities in patients suffering from cancer, infectious diseases or autoimmune disorders. These neutrophil–dendritic cell hybrids, upon exposure to cytokines such as CSF2 and IFN-γ or immune complexes, can express MHC-II and co-stimulatory molecules and function as antigen-presenting cells, activating both CD4^+^ and CD8^+^ T cells [85,86,87]. As these antigen-presenting neutrophils have been found in tumor-draining lymph nodes earlier than DCs [88], it has been suggested that they can orchestrate the initial and crucial first anti-tumor responses. However, in more advanced tumor stages, because of elevated CSF2 and IFN-γ levels in the TME, they start expressing PD-L1 and acquire an immunosuppressive phenotype, leading to worse prognoses for cancer patients [85,88,89]. 

## 6. Reverse Transmigration of Neutrophils

Neutrophil clearance at inflammatory sites is essential to maintain homeostasis. The established theory in which activated neutrophils undergo apoptosis/necrosis and subsequent phagocytosis via macrophages after executing their effector functions has been modified over the last two decades. Several groups utilizing in vivo advanced imaging technologies have demonstrated that activated neutrophils show high expressions of ICAM1 and low expressions of CXCR1, a unique phenotype compared with circulatory (ICAM1^low^/CXCR1^high^) and tissue-resident neutrophils (ICAM1^high^/CXCR1^high^), are able to migrate from the peripheral organs back to the circulation [90]. The mechanisms underlying this process, known as reverse transmigration, are complex and not fully elucidated. CXCL1 leakage from the tissue into the circulation upon breach of the endothelium; damages to the endothelial junctions because of neutrophil elastase secretion triggered by LTB4; the increased expression of CXCL8, prostaglandin E2 (PGE2), lipoxin 4 (LXA4) and cathepsin C; as well as the inactivation of hypoxia-inducible factor 1 alpha (HIF-1α), are a few of the proposed mechanisms involved in neutrophil reverse transmigration (Figure 4) [90,91,92]. The biological role of this novel process remains to be determined. However, based on the timing and severity, it has been suggested to be both a protective response, such as promoting inflammation resolution, and a tissue-damaging event, leading to the dissemination of inflammation and organ failure. This diverse function has been depicted by different studies. Wang et al. demonstrated that, upon sterile hepatic injury, activated PMNs migrated from the liver to the lungs, where, via the modulation of their CXCR4/CXCL12 signaling, they eventually returned to the bone marrow to undergo apoptosis (Figure 4) [93,94]. In addition, several research groups showed that the reverse transmigration of neutrophils (induced by LTB4 or extracellular cold-inducible RNA-binding protein—CIRP) resulted in worse outcomes in sepsis in mice [95,96].

## 7. Neutrophils in Severe Infection and Inflammation

The most common, everyday function of neutrophils is combating infection. Neutrophils routinely patrol tissues for pathogens like bacteria and viruses. Upon encountering signs of microbial infection, neutrophils quickly respond to trap and kill the invading pathogens. In addition, they secrete chemokines, e.g., CXCL8, causing additional neutrophil influx in the inflamed tissue.

### 7.1. Emergency Granulopoiesis

During severe systemic inflammation, additional neutrophils are produced in a process called emergency granulopoiesis. Clinical signs of this demand-adapted hematopoiesis are blood leukocytosis, neutrophilia and the appearance of immature neutrophil precursors in the peripheral blood (also known as left-shift), caused by the enhanced de novo generation of neutrophils as a result of increased myeloid progenitor cell proliferation [97,98]. This switch from steady-state granulopoiesis to emergency granulopoiesis is mediated by a change at the transcription factor level, where CCAAT/enhancer-binding protein (C/EBP)β takes over from C/EBPα, accelerating the cell cycle progression of myeloid progenitors and increasing neutrophil production [99]. Several cytokines have been associated with emergency granulopoiesis, of which CSF3, CSF2 and IL-6 are the best studied. CSF3 is not only essential for steady-state granulopoiesis but also plays a key role in emergency granulopoiesis, as indicated by increased CSF3 levels in patient sera upon severe infection and the fact that the administration of CSF3 accurately mimics the physiological responses observed during emergency granulopoiesis [100,101]. In addition to CSF3, CSF2 and IL-6 are shown to play an important role in emergency granulopoiesis [102]. *Csf2^−/−^* mice show normal steady-state hematopoiesis, but upon *Listeria monocytogenes* and *Mycobacterium avium* infection, they present with severe depletions in hematopoietic cells in the bone marrow and a deficient inflammatory response in infected tissues [103,104]. IL-6-deficient mice have been shown to be more susceptible to *Candida albicans* infection [19]. In addition, in mice that lack both *Csf3* and *Csf2*, IL6 trans-signaling was shown to be important, and the additional knockout of this third cytokine resulted in a 50% further decrease in granulopoiesis in vitro [20].

### 7.2. Neutrophils in Autoimmune Diseases

In contrast to their protective function against pathogens, increasing evidence links an abundance of pro-inflammatory neutrophils to the pathogenesis of several autoimmune diseases like multiple sclerosis (MS); systemic lupus erythematosus (SLE); rheumatoid arthritis (RA); type I diabetes; and inflammatory bowel diseases, including Crohn’s disease and ulcerative colitis [98,105]. In mouse models of MS, ROS and the azurophilic granule protein myeloperoxidase (MPO) were shown to destruct the blood–brain barrier and damage tissue [106,107,108]. In addition, central nervous system (CNS)-infiltrating neutrophils were shown to secrete IL-6, IL-12, IFN-γ and TNF-α, resulting in dendritic cell maturation, which subsequently activated myelin-specific T-cells, considered to be the initiating event in MS pathology [109]. Neutrophils are an important source of TNF-α and BAFF in RA, involved in the recruitment of T and B cells, respectively [110,111]. Moreover, neutrophils participate in the destruction of cartilage by stimulating the release of MMPs, while the activation of osteoclast via RANKL signaling results in bone resorption [112,113,114]. In type I diabetes, ROS, IL-1, TNF-α and IFN-γ produced by neutrophils participate in the initiation of pancreatic β-cell destruction [115,116]. In addition, NETs have been shown to contribute to pathological processes in SLE and RA, inducing endothelial damage and the externalization of citrullinated autoantigens and immunostimulatory molecules, respectively [117,118].

## 8. Neutrophils in Tumor Tissue

**Figure 5:**
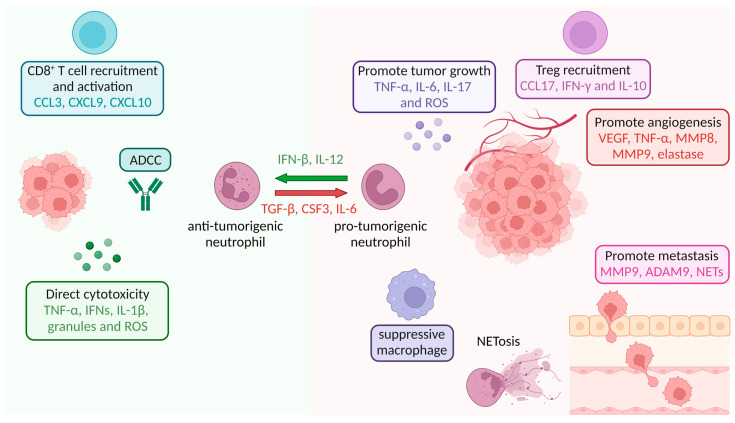
Anti- and pro-tumorigenic neutrophils in tumor tissues. Neutrophil priming occurs upon cytokine stimulation and results in either anti- or pro-tumorigenic neutrophil populations. Anti-tumorigenic neutrophils can kill tumor cells via direct cytotoxicity or ADCC and attract other immune cells like CD8^+^ cytotoxic T cells. In contrast, pro-tumorigenic neutrophils attract suppressive immune cells, e.g., suppressive macrophages and regulatory T cells (Tregs); form NETs; and promote tumor growth, angiogenesis and metastasis.

Classical views of neutrophils in cancer define them as either anti- or pro-tumorigenic, also known as N1 or N2 tumor-associated neutrophils (N1 and N2 TANs), respectively (Figure 5). Pro- and anti-tumorigenic neutrophils can be distinguished based on their cytokine repertoire. Anti-tumorigenic neutrophils produce cytokines that can promote CD8^+^ T cell recruitment and activation, e.g., CCL3, CXCL9 and CXCL10, as well as pro-inflammatory cytokines like IL-12, TNF-α and CSF2 (Figure 5) [119,120]. On the other hand, pro-tumorigenic neutrophils upregulate CCL2, CCL3, CCL4, CCL8, CCL12, CXCL1, CXCL2, CXCL8 and CXCL16 and attract CD4^+^ regulatory T cells (Tregs) by secreting high levels of CCL17 (Figure 5) [121,122].

A transition from N1 to N2 TANs can occur and is described as being regulated by TGF-β, while CSF3 and IL-6 have also been linked to inducing a pro-tumorigenic neutrophil phenotype (Figure 5) [119,123]. In contrast, IFN-β treatment, as well as IL-12, are associated with a transition toward anti-tumorigenic N1 neutrophils (Figure 5) [124,125,126]. Among the described anti-tumorigenic functions of neutrophils are direct cytotoxicity (via the release of a combination of ROS, granule contents and cytokines, such as TNF-α (by binding to TNFR1), IFNs and IL-1β,) and antibody-dependent cellular cytotoxicity (ADCC). Pro-tumorigenic neutrophils have been shown to directly promote tumor growth via cytokine secretion (e.g., TNF-α (by binding to TNFR2), IL-6 and IL-17) and ROS production (thereby increasing mutagenesis); form NETs; recruit tumor-supporting cells into the TME; promote angiogenesis; and induce tumor cell motility, migration and invasion (Figure 5) [121,127,128,129]. TNF-α can exert both anti- and pro-tumorigenic functions, depending on which of the two receptors it interacts with [130,131]. Upon binding to TNFR1, TNF-α induces pro-inflammatory responses; activates NF-κB and MAPK signaling; and induces apoptosis. On the other hand, TNF-α can induce cellular transformation, survival, proliferation, invasion, angiogenesis and metastasis by binding to TNFR2 [131]. In addition, several studies have shown that neutrophils can exert immunosuppressive functions, terming them polymorphonuclear myeloid-derived suppressor cells (PMN-MDSCs). Whether these PMN-MDSCs are a distinct neutrophil subset remains a topic of debate, which will be discussed in more detail later in this review.

On top of the pathogen/tumor cell killing effect of granules described above, several granule contents are associated with pro-tumorigenic effects. The serine proteases present in the azurophilic granules, such as neutrophil elastase and cathepsin G (Figure 1), are described as promoting tumor proliferation and/or invasion (Figure 5) [132,133,134]. By remodeling basal membranes and extracellular matrices, the specific granule protein neutrophil collagenase, also known as matrix metalloproteinase-8 (MMP-8), and the gelatinase granule proteins MMP9/gelatinase B and ADAM9 promote neutrophil infiltration and angiogenesis (Figure 5) [135,136,137]. Additionally, NET production via neutrophils has been reported to promote the migration and extravasation of tumor cells. NETs have demonstrated their ability to capture disseminated colorectal cancer cells, subsequently triggering the production of pro-inflammatory cytokines, including CXCL8, IL-6 and TNF-α. This cytokine storm led to augmented neutrophil recruitment and increased NET formation, establishing a detrimental cycle that connects NETs with the inflammatory microenvironment in liver metastasis in colorectal cancer [138]. In addition to tumor cell entrapment, NETs contain various components, including cytokines and chemokines, which can promote tumor growth and progression by attracting immune cells, promoting angiogenesis and creating a favorable microenvironment for tumor cell survival and proliferation [139].

### 8.1. Additional Neutrophil Subsets in Tumor Tissue

Recent single-cell RNA-sequencing studies have added more complexity to TANs by identifying additional sub-groups of neutrophils in the tumor tissues of treatment-naïve patients with non-small-cell lung cancer and tumor-bearing mice [26]. Five human neutrophil populations were identified (*h*N1-5), in contrast to seven distinct mouse neutrophil populations (*m*N1-6, where *m*N1 is subdivided into N1a and N1b) [26,126]. Canonical neutrophil markers like *MMP8*, *MMP9*, *ARG1*, *S100A8* and *S100A9* could be detected in both *m*N1 and *h*N1. Based on SPRING/nearest-neighbor analyses of the neutrophil subsets, the majority of other subpopulations were found to originate from these *m*N1 and *h*N1 neutrophils. Among the other subsets were the tumor-specific *m*N5 and *h*N5 neutrophils, which showed the mRNA expression of cytokines, including *CCL3*, *CSF1*, *IRAK2* and *MIF*. The *m*N2 and *h*N2 neutrophils formed a rare subpopulation, which was characterized by the expression of type I interferon response genes (e.g., *MX1*, *IFIT1*, *IRF7*). In addition, mouse neutrophil populations 4–6 (*m*N4-6) were highly tumor-enriched and showed the expression of *Siglecf*, previously linked to several pro-tumorigenic functions, including angiogenesis; extracellular matrix remodeling; the suppression of T cell responses; and tumor cell proliferation and growth [140].

### 8.2. Myeloid-Derived Suppressor Cells

PMN-MDSCs are immature suppressive neutrophils that expand under pathological conditions, e.g., inflammation and cancer, and share similarities with neutrophils, including their origin [141]. Whether they are distinct from pro-tumorigenic TANs and/or the immature LDN fraction found in the blood remains a topic of debate, especially since their terminology is used interchangeably.

In mice, PMN-MDSCs are defined as CD11b^+^Ly6G^+^Ly6C^low^ cells, while in humans, they are defined as CD14^−^CD11b^+^CD15^+^(CD66b^+^) cells, similar to neutrophils [142,143]. Human PMN-MDSCs can be separated via gradient centrifugation with 1.077 g/mL density gradient media, e.g., Histopaque, from normal neutrophils in a similar manner to LDN neutrophils (see Section 3. Circulating Neutrophils) [144]. Additionally, lectin-type oxidized LDL receptor 1 (LOX-1) has been identified as a marker to differentiate between human PMN-MDSCs and neutrophils. PMN-MDSCs, unlike neutrophils, exhibit heightened immunosuppressive properties by stimulating the production of Tregs through the secretion of IFN-γ and IL-10 (Figure 5) [145]. Additionally, they induce a suppressive M2 phenotype in macrophages (Figure 5) [146]. Moreover, PMN-MDSCs impede lymphocyte homing, potentially via the expression of the metalloprotease ADAM 17 (TACE) on the MDSC surface [147]. They also generate reactive oxygen and nitrogen species and secrete suppressive factors such as indoleamine 2.3-dioxygenase (IDO), IL-10, TGF-β and arginase-1. Notably, PMN-MDSCs deplete the microenvironment of the metabolite L-arginine, which serves as a substrate for arginase-1 and inducible nitric oxide synthase (iNOS). Consequently, T cell proliferation and activation are hindered, and the expression of the TCR-ζ chain is decreased [148]. The suppressive capabilities of circulating PMN-MDSC have been linked to unfavorable clinical outcomes across various cancer types [149,150]. Notably, the expression of arginase-1 can be increased by Th2 cytokines, including IL-4, IL-10 and IL-13 [151]. Conversely, the expression of iNOS in MDSC is primarily governed by Th1 cytokines like IFN-γ, TNF-β and TNF-α. Furthermore, Jiang et al. demonstrated the IL-6-modulated, MDSC-mediated suppression of cytokine secretion in T cells via STAT3 signaling in a breast cancer model, which could be blocked by anti-IL-6 [152]. Additionally, Park et al. demonstrated that the combination of CSF2 and stem cell factor (SCF) is the most potent enhancer for expanding and differentiating functional MDSCs from human cord blood [153]. It is noteworthy that CSF2 is produced by tumor cells and is regarded as a double-edged sword, as excessive or insufficient levels of CSF2 have been reported to promote tumor progression [154].

## 9. Effect of TME-Secreted Cytokines on Neutrophils and PMN-MDSCs

Various cellular components within the TME, such as cancer cells, stromal cells (including cancer-associated fibroblasts/CAFs) and immune cells secrete a wide array of cytokines and chemokines. These cytokines and chemokines have the ability to diffuse through the surrounding tissues, serving as potential signals to circulating or tissue-patrolling neutrophils or more immature neutrophils in the bone marrow (e.g., PMN-MDSCs and LDN), ultimately attracting them to the tumor microenvironment.

### 9.1. Effect of Cytokines on Neutrophil and PMN-MDSC Migration

The upregulation of chemokines within the TME represents the initial step in neutrophil recruitment, which is mainly regulated by chemokines like CXCL1, CXCL2, CXCL8 and their corresponding receptors, CXCR1 and CXCR2 [155,156,157,158]. In contrast, the migration of PMN-MDSCs is influenced by the chemokine receptor CCR2 and its ligands, including CCL2 and other chemokines such as CXCL5, CXCL12 and CCL3. The specific chemokines involved depend on the particular TME or inflammatory conditions (reviewed by Hao et al.) [159]. Interestingly, several studies have indicated a modulatory effect of tumor-produced factors on neutrophil and PMN-MDSC recruitment. For instance, Wu et al. reported that the IL-17/CXCR2 axis in tumor cells facilitated breast cancer progression by enhancing neutrophil recruitment [160]. Interestingly, the mRNA expression of the IL-17/CXCR2 axis players *CXCR2*, *IL17* and *IL17R* increased in Cl66, a doxorubicin- and paclitaxel-resistant murine breast cancer cell line. In addition, it was reported that breast cancer cells secrete IL-1β, resulting in IL-17 production via γδ T cells [150]. Increased IL-17 levels led to the systemic upregulation of CSF3, which subsequently caused neutrophil expansion and the alteration of neutrophils in the TME with a PMN-MDSC phenotype showing high iNOS expression. In line with this study, it was reported that activated inflammatory DCs induced γδT17 cells to secrete CXCL8, TNF-α and CSF2 with a concomitant accumulation of PMN-MDSC with high arginase-1 and ROS production in the tumor [161]. Furthermore, IL-17 is reported to increase the tumor cell expression of CXCL5, thereby enhancing PMN-MDSC infiltration in hepatocellular carcinoma (HCC) [162].

Additionally, CCL20 produced by breast cancer cells has been reported to modulate PMN-MDSC to promote cancer cell stemness through the CXCL2-CXCR2 pathway [163]. Moreover, tumor-secreted cytokines like CXCL8, PDGF, MIP1 and CSF3 increase the mobilization of neutrophils from the bone marrow and spleen, leading to an elevated neutrophil-to-lymphocyte ratio in both human [156,164,165,166] and mouse [22,167] studies. The levels of these cytokines in the circulation tend to rise as the tumor progresses.

### 9.2. Effect of Cytokines on Neutrophil and PMN-MDSC Polarization

Several studies have indicated the modulatory roles of cytokines in the phenotypes of neutrophils and PMN-MDSCs. For instance, type I interferons like IFN-β have been shown to increase the tumor cytotoxicity of neutrophils; increase NET, ICAM1 and TNF-α expression; and polarize TANs toward an anti-tumor N1 phenotype in vivo [124]. Cheng et al. reported that CAFs in hepatocellular carcinoma (HCC) attracted neutrophils through the CXCL12/CXCR4 pathway and sustained their survival and activation via IL-6-induced JAK-STAT3 signaling [168]. Neutrophils primed by HCC-CAFs exhibited increased CD66b and PDL1 expression and decreased CD62L expression. These primed neutrophils suppressed T cell immunity through the STAT3-PDL1 pathway, which could be reversed by the STAT3 inhibitor S31. Moreover, TGF-β produced in the TME has been reported to drive the transition of anti-tumor N1 TAN into suppressive PMN-MDSC [22] or suppressive N2 TAN [119]. Furthermore, TGF-β production via triple-negative breast cancer and colorectal cancer cells was reported to recruit neutrophils [169,170], demonstrating that TGF-β is involved in both neutrophil migration and polarization. Furthermore, the TME frequently exhibits elevated levels of S100A9, which facilitates the chemotaxis of MDSCs and promotes their suppressive functions. This is achieved through the engagement of TLR4 and RAGE in MDSCs, thereby activating pivotal factors such as ROS, arginase-1, iNOS and IL-10 [171].

### 9.3. Effect of Cytokines on Neutrophil Recruitment into the Premetastatic Niche

Chemokines play a pivotal role in recruiting neutrophils and PMN-MDSC, not only into primary tumor sites but also pre-metastatic niches and metastatic sites. In a mouse model of breast cancer, tumor-associated mesenchymal stromal cells released CXCL1, CXCL2 and CXCL5, leading to increased neutrophil recruitment at primary tumor sites [157]. Furthermore, CXCL5 and CXCL7 released from tumor-activated platelets were identified as essential factors for neutrophil recruitment to the pre-metastatic niche, facilitating subsequent tumor cell seeding in mouse lungs [172]. Furthermore, the tumor-derived protease cathepsin C played a significant role in promoting breast-to-lung metastasis through its involvement in neutrophil recruitment and the formation of NETs [173]. Cathepsin C facilitated this process by enzymatically activating neutrophil membrane-bound proteinase 3 (PR3), leading to IL-1β processing and subsequent NFκB activation in neutrophils. Consequently, IL-6 and CCL3 were upregulated, thereby facilitating neutrophil recruitment. Additionally, GPR35^+^ MDSC colonization to the lung was promoted in a lung metastasis model of breast cancer via the tumor secretion of CXCL17 and CSF3 [111]. PMN-MDSC recruitment into the premetastatic niche was reported to rely on hypoxic cell-derived CCL2, which is often produced by hypoxic tumor cells [174].

### 9.4. Effect of Cytokines on NET Formation

Tumor cells, CAFs and immune cells have been reported to induce the formation of NETs via neutrophils through various mechanisms. In the TME, cytokines such as CXCL8, IL-1β and TNF-α have been demonstrated to stimulate the production of ROS, leading to the release of NETs [138,175,176]. This creates a positive feedback loop, amplifying the inflammatory response. Additionally, IL-17 has been reported to induce NET formation in pancreatic cancer, which mediates resistance to immune checkpoint blockade [177]. NET formation via PMN-MDSC has not been intensively studied, but studies suggest that different mechanisms are involved compared with NET formation in neutrophils. For instance, complement C5a was reported to induce the formation of NETs via PMN-MDSC in order to promote metastasis in a mouse lung metastasis model [178]. Finally, the TME has additional influences on neutrophils that are beyond the scope of this review, including extracellular matrix remodeling, hypoxia, metabolic factors and extracellular vesicles, which are thoroughly reviewed elsewhere [179].

## 10. Exploitation of Neutrophil Functions to Combat Disease

With increasing knowledge about neutrophil plasticity and function, there is growing interest in exploring new therapeutic interventions to harness neutrophils’ innate capabilities to target and eliminate pathogens and cancer cells. Such potential strategies could target neutrophil recruitment and polarity; modulate neutrophil activation; or reduce excessive inflammation. As the field of immunotherapy is continuously evolving, several innovative therapeutic approaches have been developed or are being developed and could be used to leverage the anti-tumorigenic potential of neutrophils and block excessive neutrophil-mediated inflammation in autoimmune diseases.

### 10.1. Cytokine Therapeutics

In conditions of exacerbated cytokine production, e.g., inflammatory and autoimmune disease, the inhibition of cytokine functions caused by monoclonal antibodies or receptor blockers has been successfully used in the clinic. For example, patients with rheumatoid arthritis and Crohn’s disease are effectively treated with various TNF-blocking monoclonal antibodies, while a human IL-12/IL-23 monoclonal antibody is used to treat psoriasis patients, both resulting in reduced neutrophil infiltration into affected tissues [129,180]. Cytokines can also be therapeutically administered, as is the case for, e.g., CSF3 in congenital neutropenia patients and IFN-α for hepatitis B [181,182]. However, cytokines are pleiotropic, resulting in unwanted systemic effects, and have a narrow therapeutic range because of, among other things, a short blood half-life and unfavorable tissue distribution, making cytokine therapy challenging [183]. The cytokine engineering field has progressed tremendously over the last few years because of the development of novel techniques and a better understanding of cytokine biology, making it possible to alter cytokines so that they, e.g., can bind specific receptors with a higher affinity, leading to reduced dosing and fewer off-target effects caused by binding to other receptors, as is performed for the IL-2 “superkine” (MDNA11), currently being tested in clinical trials [184]. Furthermore, the half-life of cytokines can be extended by employing polyethylene glycol (PEG), a process that increases the molecular weight of the protein. This modification reduces renal clearance, protecting cytokines from degradation due to proteolytic enzymes and reducing their interaction with plasma constituents, thereby diminishing immunogenicity [185]. Another strategy often used to circumvent the limitations of cytokine drugs is the creation of synthetic cytokines (synthekines) using computational tools, overcoming things like pleiotropy, redundancy, poor pharmacokinetics and toxicity [186]. Multiple engineered cytokines are currently in clinical trials, as reviewed by Deckers et al. [187].

### 10.2. Immunocytokines

Genetically fusing a cytokine to another protein can help reshape the cytokine’s biodistribution profile, overcome poor pharmacokinetic properties and help promote tumor localization. This application is especially interesting in cancer, where a cytokine can be fused to a therapeutic antibody, specifically recognizing a tumor-associated antigen. These fusion constructs, called immunocytokines, hold promise as potential treatments and are currently undergoing evaluation in clinical trials [187]. For example, the CD38–IFNα2b immunocytokine TAK-573 is being tested in a phase I/II clinical trial for refractory multiple myeloma. Despite their potential, some immunocytokines have a lot of side effects because of the off-target binding of the cytokine to its receptor, resulting in the so-called “sink effect”, requiring high doses of the drug. Several engineering strategies are being developed to make the cytokine only active when it is near the tumor, one of which is Orionis Biosciences’ Activity-on-Target cytokines (AcTakines). These AcTakines are engineered to have a reduced receptor affinity, hampering cytokine activity until the immunocytokine accumulates near a target cell [188].

### 10.3. Immunotherapy

In addition to the cytokine part of immunocytokines, the antibody itself can also affect neutrophils by initiating neutrophil-mediated tumor cell killing via ADCC. All antibodies used for immunotherapy purposes are of the IgG isotype, which can bind various Fc gamma receptors on immune cells. Human neutrophils express the activating Fc-gamma receptors FcγRI (CD64), FcγRIIa (CD32a) and FcγRIIIa (CD16a) [189,190]. In addition, they also express the inhibitory receptor FcγRIIb (CD32b) and the GPI-linked and, therefore, signaling dead, receptor FcγRIIIb (CD16b), of which the latter is by far the highest expressed FcγR in neutrophils [189,190]. Therefore, IgG antibodies are not very efficient in engaging neutrophils in tumor cell killing by themselves. However, a recent in vivo study showed the effective, neutrophil-mediated killing of B16 melanoma cells when combining an IgG antibody targeting gp75 (a protein expressed on B16 melanoma cells), an CD40 antagonist and TNF [191]. The findings indicated that a combination of all three components was necessary for successful tumor cell killing. This suggests that a multimodal approach combining immunotherapy with cytokine therapy could hold great potential for engaging neutrophils in tumor cell killing and could contribute to the development of novel strategies for cancer treatment.

In contrast to IgG antibodies, IgA antibodies strictly bind the activating FcαR (CD89), making them very efficient in activating neutrophils and inducing ADCC [189]. However, IgA antibodies have a short half-life because of fast clearance via the asialoglycoprotein and mannose receptors, recognizing the extensive glycosylation of IgA antibodies [192,193,194]. In addition, IgA antibodies lack a binding site for the neonatal Fc receptor (FcRn), which recycles IgG antibodies, thereby contributing to the short half-life compared with IgG antibodies [195]. The antibody engineering of IgA has been described and resulted in an IgA3.0 molecule with an increased stability and half-life, overcoming some major hurdles of IgA immunotherapy [196,197]. In addition to being effective in activating neutrophils from healthy donors and mice, preliminary data suggest that suppressive neutrophils are as capable as normal neutrophils in killing tumor cells with IgA antibodies, making them ideal candidates to induce all neutrophil subsets to kill cancer.

## 11. Conclusions and Perspectives

The identification of different neutrophil subsets and the dual role of neutrophils in cancer have shifted the field of neutrophil biology tremendously. Gaining a comprehensive understanding of neutrophil functions in tissues in various tissue states (steady state, inflammation, cancer), as well as their plasticity and their role in the TME, is essential for harnessing their anti-tumorigenic potential effectively. The identification of cytokines that polarize neutrophils toward an anti-tumorigenic phenotype, e.g., IFN-β or IL-12, as well as cytokines that promote a pro-tumorigenic phenotype, e.g., TGF-β, has been essential for understanding the effect of the TME on neutrophil plasticity and opened up possibilities for cytokine therapy. The field of bioengineering has made remarkable advancements, leading to the study and in vivo or clinical trial testing of synthekines, immunocytokines and IgA antibodies as potential anti-cancer therapies. To facilitate the transition from pro-tumorigenic neutrophils to anti-tumorigenic neutrophils that effectively combat cancer cells, immunocytokines containing IFN-β or IL-12, known to induce the transition to N1 neutrophils (Figure 5), should be investigated. Moreover, attracting additional “naïve” neutrophils that are not pro-tumorigenic to the tumor site using immunocytokines containing CSF3, IL-1α, IL-1β or TNF-α holds immense potential in enhancing (IgA) immunotherapy and optimizing the anti-tumorigenic capabilities of neutrophils.

## Data Availability

Not applicable.
